# Maturation of the oral microbiota during primary teeth eruption: a longitudinal, preliminary study

**DOI:** 10.1080/20002297.2022.2051352

**Published:** 2022-03-16

**Authors:** He Xu, Bijun Tian, Weihua Shi, Jing Tian, Wenjun Wang, Man Qin

**Affiliations:** Department of Pediatric Dentistry, Peking University School and Hospital of Stomatology & National Center of Stomatology & National Clinical Research Center for Oral Diseases, Beijing, Hebei Province, China

**Keywords:** Saliva, plaque/plaque biofilms, microbiota, growth/development, cohort studies

## Abstract

**Introduction:**

Oral microbiota that established in the early years of life may influence the child’s oral health in the long term. Until now, no consensus is reached about whether the development of the oral microbiota is more related with age increase or more with teeth eruption.

**Objective:**

To analyze the microbiota development of both saliva and supragingival plaque during the gradual eruption of primary teeth in caries-free infants and toddlers.

**Methods:**

Saliva and plaque samples were collected at five and four dentition states, respectively, and were identified by bacterial *16S rRNA* gene sequencing.

**Results:**

During the longitudinal observation, the saliva ecosystem seemed more complex and dynamic than the plaque, with larger bacteria quantity and more significantly varied species over time. About 70% of the initial colonized OTUs in plaque persisted until the completion of the primary dentition. Transient bacteria were mostly detected in the early saliva and plaque microbiota, which came from the environment and other sites of the human body. Microbial diversity in both saliva and plaque varied greatly from pre-dentition to full eruption of eight anterior teeth, but not during the eruption of primary molars.

**Conclusion:**

Oral bacterial development follows an ordered sequence during the primary teeth eruption. ‘Fully eruption of all primary anterior teeth’ is a critical stage in this process.

## Introduction

The human oral cavity is a complex ecosystem that comprises hundreds of bacterial taxa and other microorganisms that interact with each other constantly. It is widely accepted that the oral microbial composition is both age and niche dependent [1]. Previous studies stated that the maturation of the oral microbiota is driven by biological changes with age [2], and the oral microbiota that established in the early years of life may influence the child’s oral and systemic health in the long term [3]. However, it was not until the last three years that several cohort studies were published, which focused on the development of the oral microbiota with aging, based on high throughput sequencing results. These studies indicated that microorganisms colonized in the infant’s oral cavity in a timely manner, with increasing complexity with time [4]; and the most distinct evolution might take place before 18 months after birth [5,6]. However, we find that the month-age span between adjacent time points in some studies was too long to elaborately represent the microbiota characters in distinct stages during the primary dentition establishment. Besides, in some studies, some participants developed caries during the longitudinal observation, but the caries children were not distinguished from the other caries-free participants in the data analysis. This might reduce the applicability of the final results to other populations in other territories. Most importantly, almost all the previous studies defined different development stages of the oral microbiota simply according to month ages, but ignored the simultaneous state of teeth eruption.

Generally, the primary incisors begin to erupt at around 6 months after birth, and primary molars can erupt as early as 12 months after birth; until around 3 years of age, all the primary molars would have erupted and a stable dental occlusion would have been established in most children. However, just like the heterogeneity in the oral microbial composition, teeth eruption time varies drastically among different individuals. For instance, a normally developed 12-month-old infant may already have four primary incisors erupted, or may still be edentulous. Until now, no consensus is reached about whether the development in the oral microbiota is more related with increase in age or more with the state of teeth eruption.

Based on this, our hypothesis was that the oral microbiota develop in an ordered sequence that relate with the gradual eruption of primary teeth. The purpose of this study was to longitudinally follow caries-free infants and toddlers at five representative dentition states, in order to characterize the development of healthy salivary and supragingival plaque microbiota during the process of primary dentition establishment.

## Materials and methods

### Ethics statements

The Ethics Committee of the Peking University School and Hospital of Stomatology Peking University Health Science Center (PKUSSIRB-201414056) approved the study design, protocol, and informed consent procedure. Written informed consents were received from parents of all the participants prior to enrollment. This study protocol was registered on China’s domestic medical research registration platform, which is subordinate to the National Health Commission of China (Registration number: MR-11-20-002849). This study was carried out according to relevant institutional guidelines and regulations.

### Participants recruitment and follow up

**Figure 1.**
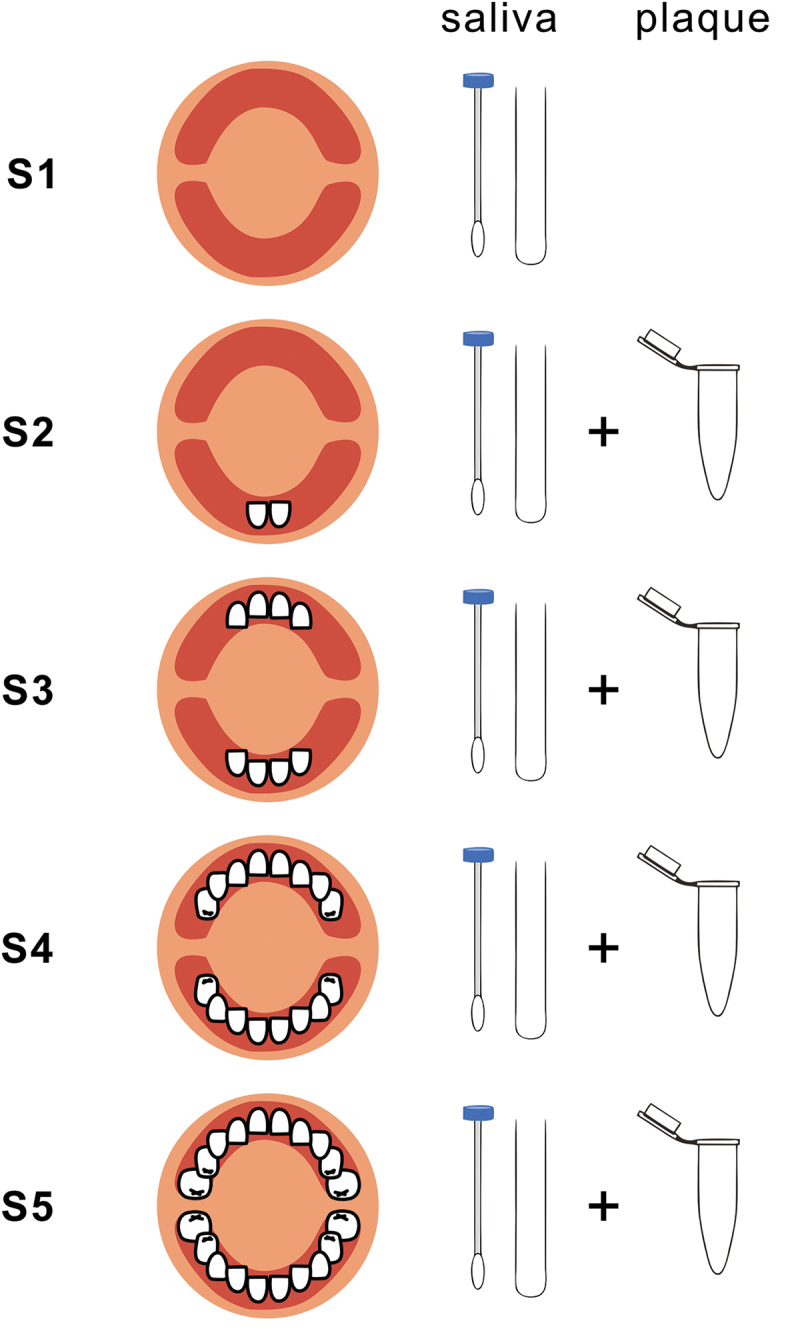
Flowchart of sampling in this study. **S1**: Infants are around 4–5 months old and with no teeth eruption. Saliva samples are obtained. **S2**: Infants are around 6 months old, with only two lower primary central incisors erupted. **S3**: Infants are around 9 months old, with all the upper and lower primary incisors (8 teeth) erupted. **S4**: Toddlers are around 14–19 months old, with all the first primary molars (16 teeth) erupted. **S5**: Toddlers are around 24–36 months old, with all the second primary molars (20 teeth) erupted. Both saliva and supragingival plaque samples are obtained at each dentition state from S2 to S5.

This was a longitudinal cohort study of infants and toddlers from about four-months-old to about 36-months-old. Twenty-one infants who were born in Beijing, China, between May 2014 and March 2015 were initially recruited. The inclusion criteria were healthy infants with full-term birth, weight up to standard, around 4 months old with no erupted deciduous teeth, no systemic diseases, no salivary gland diseases, no history of antibiotic use within the previous one month. Saliva (recruited to the B group) samples were taken from these infants at five particular dentition states (S1 to S5), while supragingival plaque (recruited to the P group) samples were taken at four dentition states (S2 to S5), all under the consents from their parents or guardians. All the sampling was ended by June 2017 (Figure 1).

Before implements of the longitudinal observation, a clear and concise training was given by one attending pediatric dentist to each participant’s parents, including the general order and month age of primary teeth eruption, the identification of each sampling dentition state, how to observe and differentiate the oral health status, illustration of sampling methods, and most importantly, oral health instructions about how to help their babies maintain oral health at different dentition states. During the longitudinal observation, the dentist kept in touch with all the parents to follow up the participant’s health and oral condition, so as to monitor the time of sample collection.

At each dentition state, the sampling was postponed if conditions of the infant/toddler were as follows: suffering from systemic infectious diseases, such as respiratory tract infection; suffering from infection in the oral cavity, such as thrush; intake of antibiotics within the previous month; or had fluoride application within the previous two weeks. Under these conditions, the sampling was restored if the participant’s oral condition still met the particular dentition when he/she regained his/her health or relieved from the wash out period. However, if any new tooth had erupted during this period and had caused the participant’s oral condition no longer to meet the required condition, the sampling was dismissed at this particular dentition state. In addition, participants would be eliminated from the cohort if caries was detected (ICDAS ≥ 2) at any time during the longitudinal observation. With these participants, all previous samples were dismissed [7]. Accordingly, all the saliva and plaque samples were taken under healthy oral conditions, with no caries or other interference.

### Oral examination and sample collection

Oral examination of all the participants was performed by one pediatric dentist under natural light. Consistency in the oral examination for this particular dentist was ensured by comparing with two other attending dentists prior to the initiation of the study. The κ value for intra-examiner agreement in the diagnosis of caries was 0.89.

Parents of the participant were asked to keep their babies away from eating or drinking for 2 h before sample collection. Oral samples were collected by the same pediatric dentist with the help of one assistant and the parents. First, saliva samples were collected using a sterile cotton swab to dip in the salivary pool at the mouth bottom and gently wipe the buccal mucosa, letting saliva to soak the swab. Then, a supragingival plaque sample was collected from all the erupted teeth using a sterilized disposable micro applicator. The tip of each swab and applicator was then cut off using sterilized scissors and transferred to a sterile 1.5 ml centrifuge tube containing 1 mL TE buffer (50 mM Tris-HCl, 1 mM EDTA; pH 8) [^8–10^]. Both the saliva and plaque samples were frozen in a − 20°C refrigerator immediately and transferred to −80°C within 1 week.

### DNA extraction and Illumina HiSeq PE250 sequencing

Microbial DNA was extracted using the QIAamp DNA Mini Kit (Qiagen Germany) according to manufacturer’s protocols, and then evaluated using 1% agarose gel electrophoresis and a NanoDrop 8,000 spectrophotometer (Thermo Fisher Scientific, Waltham, MA). Based on the quantity and quality of isolated DNA, a total of 26 saliva samples and 25 supragingival plaque samples were selected for sequencing. The V3-V4 region of the *16S ribosomal RNA* genes was amplified by PCR (95°C for 3 min, followed by 30 cycles at 98°C for 20 s, 58°C for 15 s, and 72°C for 20 s, with a final extension at 72°C for 5 min) using the primers 341 F (5’-ACTCCTACGGGRSGCAGCAG-3’) and 806 R (5’-GGACTACVVGGGTATCTAATC-3’). Amplicons were extracted from 2% agarose gels and purified using the AxyPrep DNA Gel Extraction Kit (Axygen Biosciences, Union City, CA) according to the manufacturer’s instructions and quantified using Qubit®2.0 (Invitrogen, MA). All quantified amplicons were pooled to equalize the concentrations and then sequenced on the Illumina HiSeq PE250 platform (Illumina, Inc., CA). The paired end reads of 250 bp were overlapped on their 3 ends for concatenation into original longer tags by using PANDAseq (https://github.com/neufeld/pandaseq, version 2.9) [11].

### Bioinformatics and statistical analyses

Assembled tags, trimmed for barcodes and primers, were further checked on their rest lengths and average base quality. To obtain high-quality reads, sequences which average quality values below 20, contained more than three ambiguous base calls (N), lengths <220 bp or >500 bp were all removed. The copy number of the tags was assessed and redundancy of repeated tags was removed. Only the tags with a frequency more than 1, which tend to be more reliable, were clustered into OTUs, each of which had a representative tag. Operational Taxonomic Units (OTUs) were clustered with 97% similarity using UPARSE (http://drive5.com/uparse/) and chimeric sequences were identified and removed using Usearch (version 7.0.1090) [12]. The expanded Human Oral Microbiome Database (eHOMD, v15.22) was used to assign sequences to specific microbial taxonomies [13].

The OTU profiling table and α diversity analysis were achieved by python scripts of QIIME (version 1.9.1). The core microbiota was identified according to the taxonomy represented by the OTUs shared by all samples. Venn diagrams were created using the VennDiagram in R to describe the number of OTUs shared among groups and unique to each group. Α diversity analysis was performed including a richness index (Chao1), a phylogenetic diversity index (PD whole tree), and diversity indexes (Shannon and Simpson) [14]. Intra-group comparison was made by the wilcox.test of R between two groups and the Kruskal test of R among more than two groups. Bray-Curtis distance matrices were calculated between samples to investigate similarities or differences in the microbiota community composition, with *P* < 0.05 considered to be a significant difference. Non-parametric multivariate analysis of variance (PERMANOVA analysis) was performed using the vegan package in R, with a *P*-value correction applying the Bonferroni method.

At the genus and species level, intra-group differences in both the saliva and plaque groups were analyzed using the Kruskal–Wallis test, with a *P*-value correction by the Bonferroni method. At each state, between-group comparison was done by the Wilcoxon method. Dentition state-discriminatory bacteria taxa (DDT) were calculated between every two adjacent dentition state in each group using the randomForest package in R. The number of DDT for a certain time period was determined using the rfcv function (10-fold cross validation).

## Data availability

The raw sequencing data are available in the NCBI BioProject database under accession number PRJNA770071.

## Results

During the longitudinal observation, two participants suspended sampling due to caries detection and another six participants dropped out due to various reasons (the missing rate was 38%). Ultimately, 13 participants completed the sampling procedure with a total of 73 samples obtained. Twenty-two samples with low DNA concentration or DNA degradation were eliminated during the quality inspection of sequencing and 51 samples were finally subjected to *16S rRNA* gene sequencing, including 26 saliva samples and 25 supragingival plaque samples. The average month age of the sequencing samples at S1-S5 was 5, 8-, 16-, 22- and 33-months-old, respectively (Appendix Table 2).

A total of 2,760,003 high-quality reads were generated. On average, 54,118 reads per sample were obtained (Appendix Table 2). The bacteria were classified into 10 phyla, 24 classes, 40 orders, 65 families, 110 genera and 215 species. All the samples were dominated by six phyla, including *Actinobacteria, Bacteroidetes, Firmicutes, Fusobacteria, Proteobacteria*, and *Saccharibacteria* (TM7) (Appendix Figure 5, Appendix Table 3).

### Variation in microbial composition in saliva over time

**Figure 2.**
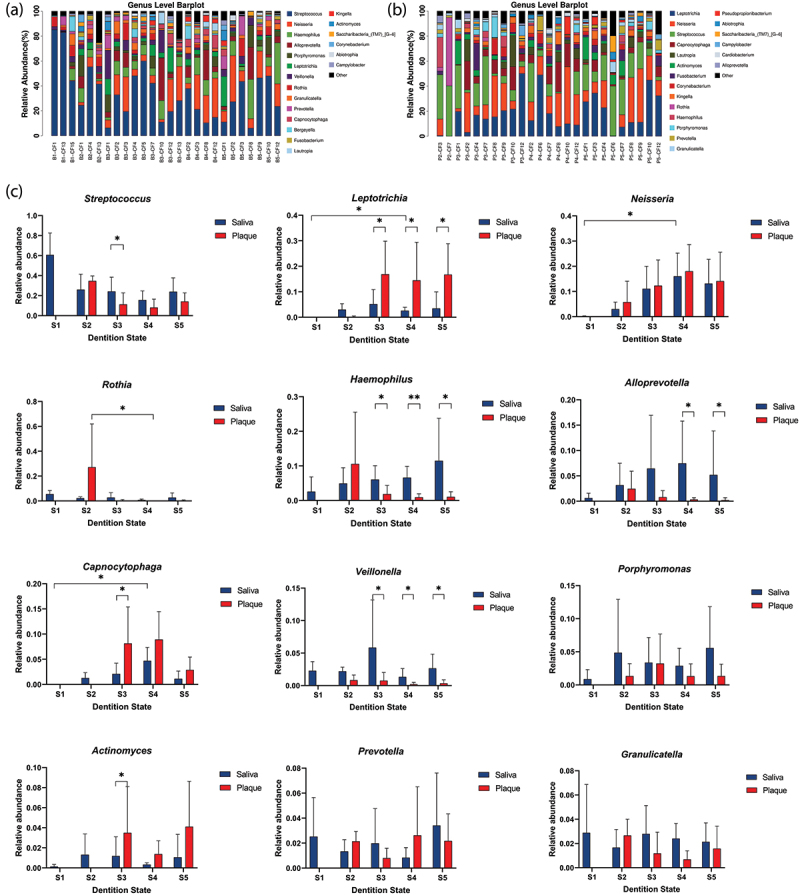
The community structure and relative abundance variation at the genus level. Taxonomic distribution of the relative abundance of major genera in saliva (a) and supragingival plaque (b) samples. Each column represents the relative abundance of microbial components in a single sample. (c) The relative abundances of major bacterial genera in saliva (blue) and supragingival plaque (red) samples over time. Significant differences are indicated by asterisks: *P < 0.05, **P < 0.01. Differences over time within the respective sample type is analyzed using the Kruskal–Wallis test, with *P*-value correction by the Bonferroni method. At each time point, between-group comparison is done by the Wilcoxon method.

The genera quantity in saliva (47–58 genera) was relatively stable and was larger than that in supragingival plaque (36–60 genera) before S5 (Figure 2). The top three genera in saliva were *Streptococcus, Neisseria* and *Haemophilus*; while the top three genera in plaque were *Streptococcus, Leptotrichia*, and *Neisseria* (Appendix Table 4).

At S1, when the infants were pre-dentate, *Streptococcus* constituted over 60% of the total microbiota, then sharply declined to 26.1% at S2 and remained relatively stable until S5. *Leptotrichia, Neisseria, Haemophilus, Capnocytophaga, Alloprevotella, Veillonella* and *Porphyromonas* increased markedly from S1 to S3 (Figure 2 and Appendix Table 4).

**Figure 3.**
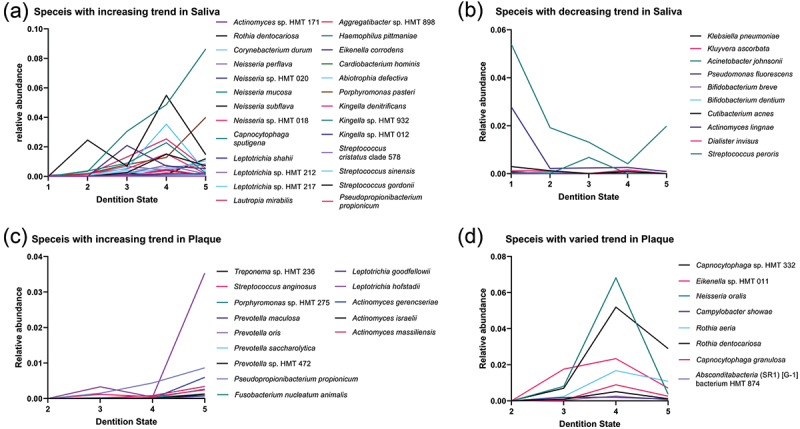
Relative abundance of bacterial species with significant variation over time in saliva and plaque. Species with significant variation over time (*P* < 0.05) that could be detected from all the sampling dentition states are shown. Species with (a) increasing and (**b**) decreasing trend in saliva over time. Species with (c) increasing and (d) varying trend in plaque over time. Intra-group differences in both saliva and plaque groups are analyzed using the Kruskal–Wallis test, with *P*-value correction by the Bonferroni method.

Of all the species that varied significantly in saliva during the teeth eruption process, some transient species were only detected at S1-S2 and rarely detected afterwards with low abundance (<0.01%), which included the following four patterns: (1) parturition-related bacteria, such as *Lactobacillus iners, Lactobacillus gasseri*, and *Prevotella bivia*, all of which were frequently isolated in the vagina [15,16];, (2) environmental bacteria, such as *Bifidobacterium subtile, Agrobacterium tumefaciens, Stenotrophomonas maltophilia*, and *Microbacterium ginsengisoli*, which used to be isolated from sewage, soil and water [17,18];, (3) resident bacteria in other sites of the human body, such as *Corynebacterium tuberculostearicum* from human skin, *Staphylococcus auricularis* from ear, as well as other species including *Corynebacterium afermentans, Anaerococcus octavius*, and *Helicobacter pylori* [19,20];, and (4) gut bacteria, such as *Lactobacillus reuteri* clade938, *Enterococcus faecalis, Enterobacter hormaechei, Bifidobacterium longum*, and *Klebsiella aerogenes*. Among the species that could be detected in saliva from all states of S1-S5, most of the increasing species were proved as components of the normal oral microbiota, while several decreasing species were reported to be frequently isolated in other sites of the human body or passed from the mother through breastfeeding [21] (Figure 3).

### Variation in microbial composition in supragingival plaque over time

When supragingival plaque were first obtained at S2, genera such as *Streptococcus, Rothia* and *Haemophilus* constituted about 72.5% of the total abundances. This total proportion sharply declined to 13.5% at S3, with substantial decrease of all these three genera. Several genera increased markedly from S2 to S3, including *Leptotrichia, Neisseria, Capnocytophaga, Actinomyces* and *Corynebacterium*. From S3 to S5, mild fluctuation persisted in the plaque genera composition which was relatively stable compared to the S2-S3 period (Figure 2 and Appendix Table 4).

Similar to the longitudinal changes in saliva, some species in the plaque microbiota were detected only at S2 or with low abundance afterwards (<0.01%), which included (1) resident bacteria in other sites of the human body, most of which came from the human skin, such as *Corynebacterium coyleae, C. tuberculostearicum* and *Cutibacterium acnes*, and other species such as *Corynebacterium tuscaniense* that used to be isolated from human blood [22];, (2) opportunistic pathogens that were occasionally detected in humans, such as *Moraxella osloensis* and *Staphylococcus schleiferi* [23];, (3) several other species, such as *Kocuria rhizophila* that relates with the environment and *Streptococcus vestibularis* that uses to be isolated from the vestibular mucosa of the human oral cavity [24,25].

Most of the species that significantly varied in plaque over time were steadily detected from S3. These species gradually became stable components of the normal plaque microbiota (Figure 3). Intriguingly, a few species were detected after the S4 state. *Treponema* sp. HMT 236 was detected only after S4, while *Treponema* sp. HMT 237, *Olsenella* sp. HMT 807, and the *Saccharibacteria* (TM7) [G-1] bacterium HMT 869 were detected only in the S5 state. Several other species exhibited markedly increased abundance or detection rate from the P4 to the P5 period, including *Leptotrichia hofstadii, Actinomyces gerencseriae* and *Actinomyces israelii*. These species may be related to the oral condition variation that associated with the eruption of second primary molars.

### Core microbiota and dentition state-discriminatory taxa (DDT) over time

The OTU composition in saliva was larger than in plaque until S4, while the overlapping OTUs between saliva and plaque increased from 23% at S2, to around 43–60% after S3. Within each sampling group, around 30% of the OTUs in saliva and 70% of the OTUs in plaque could be detected through all the dentition states indicating that about 70% of the initially colonized OTUs in plaque would persist through the eruption process of all primary teeth (Appendix Figure 6 and 7).

Six OTUs were shared across all the samples at all dentition states, i.e. *Neisseria mucosa, Veillonella dispar*, and four species of *Streptococcus* (*S. cristatus* clade 578, *S. gordonii, S. lactarius* and *S. oralis* subsp. clade 398). Another 10 OTUs were detected from all the saliva samples; *Alloprevotella* sp. HMT 473, *Bergeyella* sp. HMT 322, *Haemophilus parainfluenzae, Gemella bergeri, Gemella haemolysans, Gemella morbillorum, Streptococcus peroris, Streptococcus sinensis, Veillonella atypica* and *Veillonella* sp. HMT 780. Another three OTUs were detected from all the plaque samples; *Capnocytophaga sputigena, Granulicatella adiacens* and *Granulicatella elegans*. Dentition state-discriminatory species were analyzed within each group. Before S3 in both groups, about half of the DDT species could be recognized as resident bacteria that originated from other sites of the body or external environment, such as *C. tuberculostearicum, Actinomyces lingnae, A. octavius, M. ginsengisoli*, and *Ottowia* sp. HMT 894 from S1-S2 in saliva; *Eggerthella lenta* and *Klebsiella pneumoniae* from S2-S3 in saliva; as well as *Streptococcus thermophilus* and *C. tuscaniense* from S2-S3 in plaque. Most of these exogenetic species declined with the gradual eruption of primary teeth, and could only be rarely detected or existed with low abundance after S3. On the contrary, most of the DDT from S3 to S5 could be recognized as common bacteria that colonized in the oral cavity (Table 1).

**Table 1.** Dentition state-discriminatory bacteria taxa in each group at the species level

Dentition state segment (age)	Saliva group	Supragingival plaque group
S1-S2 (5 m-8 m)	*Corynebacterium tuberculostearicum*	
	*Lactobacillus gasseri*	
	*Lautropia mirabilis*	
	*Actinomyces lingnae* [Not validly published]	
	*Anaerococcus octavius*	
	*Leptotrichia* sp. HMT 225	
	*Leptotrichia* sp. HMT 417	
	*Haemophilus parainfluenzae*	
	*Neisseria subflava*	
	*Neisseria weaveri* *Ottowia* sp. HMT 894 *Microbacterium ginsengisoli*	
S2-S3 (8 m-16 m)	*Leptotrichia* sp. HMT 212	*Corynebacterium tuscaniense*
	*Eggerthella lenta*	*Streptococcus thermophilus*
	*Granulicatella elegans*	*Campylobacter showae*
	*Klebsiella pneumoniae*	*Actinomyces* sp. HMT 170
	*Eggerthella lenta*	*Abiotrophia defectiva*
		*Neisseria elongata*
S3-S4 (16 m-22 m)	*Haemophilus pittmaniae*	*Porphyromonas* sp. HMT 275
	*Aggregatibacter* sp. HMT 898	*Rothia aeria*
	*Kingella* sp. HMT 012	*Kingella* sp. HMT 012
	*Pseudopropionibacterium propionicum*	
	*Veillonella* sp. HMT 780	
	*Streptococcus peroris*	
S4-S5 (22 m-33 m)	*Capnocytophaga sputigena*	*Saccharibacteria* (TM7) [G-1] bacterium HMT 347
	*Moraxella osloensis*	*Capnocytophaga granulosa*
	*Kingella* sp. HMT 012	*Saccharibacteria* (TM7) [G-1] bacterium HMT 869
	*Cardiobacterium hominis*	*Leptotrichia hofstadii*
	*Leptotrichia* sp. HMT 217	*Lachnoanaerobaculum orale*
	*Peptostreptococcaceae* XI [G-7] [*Eubacterium*] *yurii* subsps. *yurii* and *margaretiae*	*Streptococcus anginosus*

### Changes in microbial profiles over time

**Figure 4.**
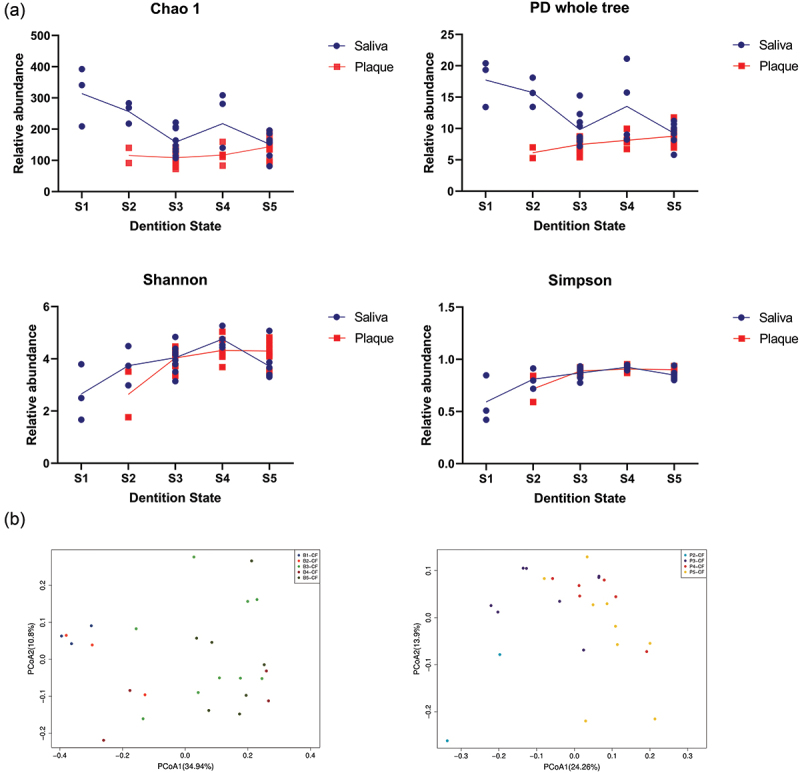
Comparison of α and β diversity of the oral microbiota over time. The α diversity of microbial profiles in saliva (blue boxes) and plaque (red boxes) of children over time. (**a**) The α diversity variation within each group in bacterial richness index of Chao1, bacterial phylogenetic diversity index of PD whole tree, and bacterial community diversity indexes of Shannon and Simpson. (**b**) Principal coordinate analysis (PCoA) plots for saliva (left) and supragingival (right) samples (principal coordinates 1 and 2) based on the unweighted UniFrac distance. Each point represents the microbiota composition of one sample. Saliva microbiota of infants in S1 (left) and plaque microbiota of infants in S2 (right) differed significantly from the older groups.

Within-group calculation showed increasing trends of microbiota diversity in both saliva and plaque. Increase in the salivary microbial diversity was detected from S1 to S4 (*P* < 0.05), although a short decreasing trend was detected in salivary microbial richness from S1 to S3 (no significance), which may be related to the short existence and abundance decreasing of the transient bacteria. In the meantime, the microbial diversity in plaque increased steadily (Figure 4). Permanova tests showed significant variations in β diversity from S1 to S3-S5 in saliva and from S2 to S3-S5 in plaque (*P* < 0.05, Appendix Table 5). No significant variation was found after S3 in both groups. These results indicate that by the state of all the eight primary incisors erupt, the microbiota structure of both saliva and supragingival plaque has been preliminarily established and will persist until the completion of primary dentition.

The between-group comparison verified the imparity between saliva and supragingival plaque in microbial composition and structure (Appendix Figure 8). Although the microbial richness was higher in saliva before S4, the plaque microbial diversity became significantly higher than that in saliva (*P* < 0.05) after eruption of the second primary molars (S5) (Figure 4). Permanova tests showed significant differences of β diversity in S3, S4 and S5 (S2: *P* = 1, R^2^ = 0.23; S3: *P* = 0.003, R^2^ = 0.16; S4: *P* = 0.003, R^2^ = 0.23; S5: *P* = 0.008, R^2^ = 0.14). These differences suggest that the plaque microbiota is slow to develop and is independent from that of saliva as a different physiological niche, instead of shift from the saliva microbiota on teeth eruption.

## Discussion

Infants are born edentulous. Bacterial colonization in the oral cavity can occur as early as 8–16 h after birth [26]. Previous studies indicated that the oral microbiota was generally acquired in an ordered time course. In the pre-dentate condition, the oral mucosal surfaces provide the only environment for bacterial colonization. Previous studies document that within the first 3 months after birth, infants have harbored a simple core microbial genus community in saliva that include *Streptococcus, Rothia, Veillonella, Granulicatella* and *Gemella* [4], which are defined as ‘early colonizers’. Genera such as *Prevotella, Granulicatella, Neisseria, Haemophilus* and *Rothia* are found to increase in the 3–6 month of age [3,27].

In our study, differential states were defined by teeth eruption instead of month of age. A similar colonization pattern was observed when the infants were pre-dentate at around 5 months old. *Streptococcus* dominated in saliva at all dentition states, but with decreasing abundance as teeth erupted gradually, and was accompanied by a rise in other genera. One possible explanation is that the metabolic products (such as lactic acid) derived from *Streptococcus* species from the dietary oligosaccharides in breast milk may facilitate the establishment of other oral commensal microorganisms, such as *Veillonella* [28,29]. Besides, as new bacteria keep being introduced into the oral cavity through various media, possible synergy and antagonism among bacteria lead to a more balanced microbial composition during dynamic development.

The oral environment becomes more complex with the emergence of primary teeth [30]. However, descriptions of the microbial variation during initial plaque formation are still limited. Previous studies presented plaque microbiota composition as early as 12 months after birth, but without any specific record of the corresponding teeth eruption condition [5,10,31]. This may introduce bias in the microbial composition description that comes from the dentition variation at the same age. Our study described the plaque microbiota development by four states defined by the teeth eruption condition, and advanced the investigation ahead to when the infants only had two lower primary central incisors erupted, at around eight months old. We found that *Streptococcus, Rothia* and *Haemophilus* constituted over 70% of the total abundances, which could be regarded as the main genera at the initial formation of supragingival plaque.

Fluctuation of bacteria persists and is teeth eruption-dependent. Previous studies reported that *Actinomyces, Porphyromonas, Abiotrophia* and *Neisseria* became dominant in saliva after the age of 1 year, and were defined as ‘late colonizers’ [3,32]. In our study, we detected a lot more genera that increased in abundance from pre-dentate to eruption of all the primary incisors (S1-S3) (Appendix Table 4). Moreover, we also detected multiple polytropic changes in the microbial composition. During this time, the external environment and other sites of the human body are important sources for bacteria that are introduced into the oral cavity, which participate in the initial formation of the oral microbiota. Both the longitudinal changes of bacterial species and the DDT analysis showed that some of these exogenous bacteria might be continuously detected in the oral cavity for a short period of time. But as the infants grow, these bacteria either disappear, or decrease in detection rate or abundance, to become a micro-component of the oral microbiota. Therefore, these bacteria can be defined as transient bacteria of the oral cavity. On the other hand, almost all the DDT species after S3 were components of the residential oral microbiota that steadily colonize in the mouth. This demarcation at S3 indicates that the oral microbiota has been gradually separated from the external environmental factors at this particular dentition state and has become a relatively independent habitat with its own characteristics.

The existence of transient bacteria in infancy and the toddler period indicates the miscibility and variability of the early oral microbiota, especially the early salivary microbiota; as well as the intersection of the oral microbiota with external bacteria from the environment and other sites of the body. The intrusion of these bacteria into the mouth may be related to parturition, breast feeding, and sucking fingers and toys. For instance, *Ottowia* used to be reported isolated from soil and water environments, but rarely from the human body [33,34]. In our study, the *Ottowia* sp. HMT 894 was detected from multiple dentition states in both saliva and plaque, although at low abundances. This indicates that possibilities may exist for some exogenous bacteria to persist in the oral cavity and become a minor residential component of the oral microbiota. Nevertheless, due to the limitations of bacterial 16S rRNA sequencing, the detected microbiota may contain dead bacteria and may not be confined to the active microbial community that actually functions in biological processes [35]. We suppose that further investigation is still needed to identify the practical roles of these transient bacteria in the oral microbiome.

Many longitudinal studies have documented increased oral microbial diversity with age. Based on different sampling time, the most actively increasing window in bacteria species was narrowed to 3–18 months or 6–18 months in age [6,36]. Previous studies showed that introduction of solid food and changes in the oral habitat played a major role in the diversity increase [4,27]. Neither the delivery mode nor partial breastfeeding habits until 12 months of age have an infection impact on the salivary species richness [3]. In our study, this increasing window took place from pre-dentition to the full eruption of all primary incisors (S1-S3), with corresponding month age of 5 to 16. This happened to be the duration that infants start from pure dairy feeding (breast milk or formula) to gradual addition of complementary food until they can basically eat the same solid food as adults. As infants get older, teeth eruption provides a physiological basis of chewing and promotes the intake of solid food. In return, the increase of food diversity and children’s activities not only introduce new bacterial species into the mouth, but also the sources of nutrient substrates for bacterial metabolism. All these aspects contribute to the increase of oral microbiota diversity. Our study also shows that this increasing window is critical in that, by the time of all primary incisors erupted, the diversity and structure of the oral microbiota had become relatively stable and would persist till the completion of the primary dentition.

Both the major and core microbiota should be defined based on niche and age [1]. Bacteria taxa that could persist across ages may limit to only a small portion of the whole microbiota [3,37], but the quantity of the core microbiota may increase with age [6]. In our study, the composition variation and core microbiota analysis further confirmed the existence of transient bacteria in the oral cavity. The core microbiota in saliva and plaque were both overlapping and unique. The variation trends of the OTUs in both niches and the persistence of most initially colonized OTUs in plaque confirm that the predominant oral microbiota is acquired early in an organized pattern; and once established, the early-colonizing bacteria tend to persist in the mouth [27,32].

Possible limitations of this study include the limited number of participants and samples. The sampling loss rate was high due to the short sampling window (S1-S4 states) based on dentition state and also consideration of both the childrens’ general condition and the short-term medication history. We consider it very necessary to control the covariate factors that may affect the microbial composition and variations, especially with limited samples, so as to control the quality of microbial description and analysis. On the other hand, the cotton swab method for collecting saliva would inevitably introduce some biofilm components from the mucosa, which is a common issue in sampling with young children. Therefore, the saliva microbiota group could be regarded as a combination of bacteria from both saliva and oral mucosa [6,8]. In addition, our previous study demonstrated that shift in the plaque microbiota had already taken place as early as 6 months before caries onset in primary dentition children [10]. Therefore, every participant in this study was followed for another 6 months after the S5 state to ensure that the child was still caries free.

In summary, this preliminary study recorded the dynamic variation of the oral microbiota during the primary dentition establishment. In general, bacterial colonization and microbiota maturation follow an ordered sequence that relate with teeth eruption, with a few bacteria persisting as core microbiota. ‘Fully eruption of all eight primary anterior teeth’ is an important stage for the oral microbiota development during the primary teeth eruption. Both oral microbial community composition and diversity vary greatly from pre-dentition to this state. While after this state, the composition and structure of the oral microbiota remained relatively stable until the eruption of all primary molars. This dentition-state mannered variation in saliva and supragingival plaque microbiota during the eruption of primary teeth provides a theoretical basis for the oral microbiota development under physiological state and may be useful in further study designs over potential associations with disease occurrence. Further studies can be applied with expanded population to verify the findings in microbial variation in this study, and may also focus on investigation of the possible biological functions of the transient bacteria as well as the dentition-state discriminatory species during the oral microbiota development, so as to screen out potential representative bacteria for important growth condition, as well as for disease prediction and prevention.

## Figures and Tables

**Figure A1.**
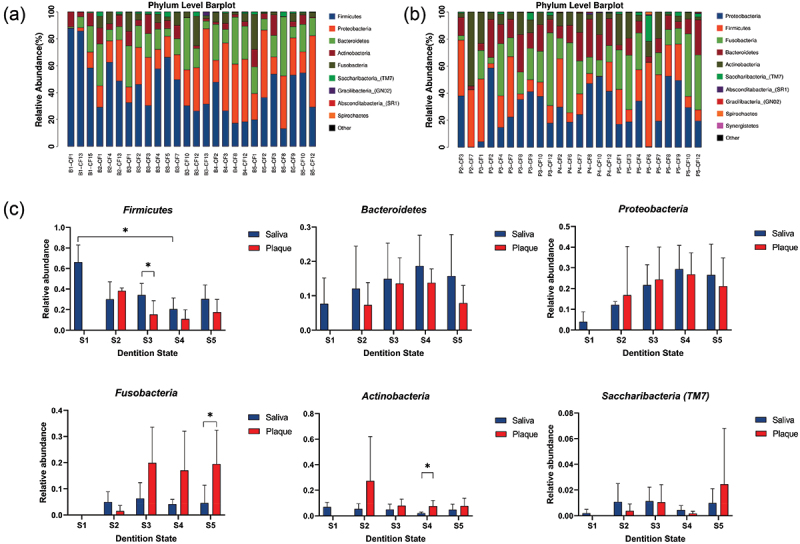
The community structure and relative abundance variation at the phylum level. Taxonomic distribution of the relative abundance of reads (%) at the phylum level in saliva (**a**) and supragingival plaque (**b**) samples. (**c**) The relative abundances variation of major bacterial phyla in saliva (blue) and supragingival plaque (red) samples over time. Significant differences are indicated by asterisks: *P < 0.05, **P < 0.01. Differences over time within the respective sample type are analyzed using the Kruskal–Wallis test, with *P*-value correction by the Bonferroni method. At each time point, between-group comparison is done by the Wilcoxon method.

**Figure A2.**
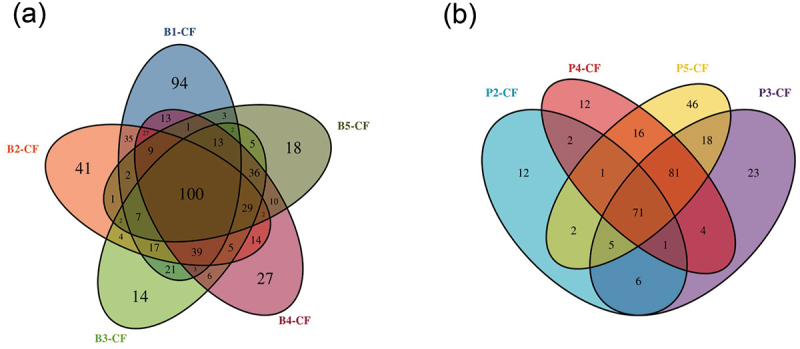
Venn diagrams depict the shared and unique OTUs within saliva (a) and supragingival plaque (b) groups over time. All OTUs are included.

**Figure A3.**
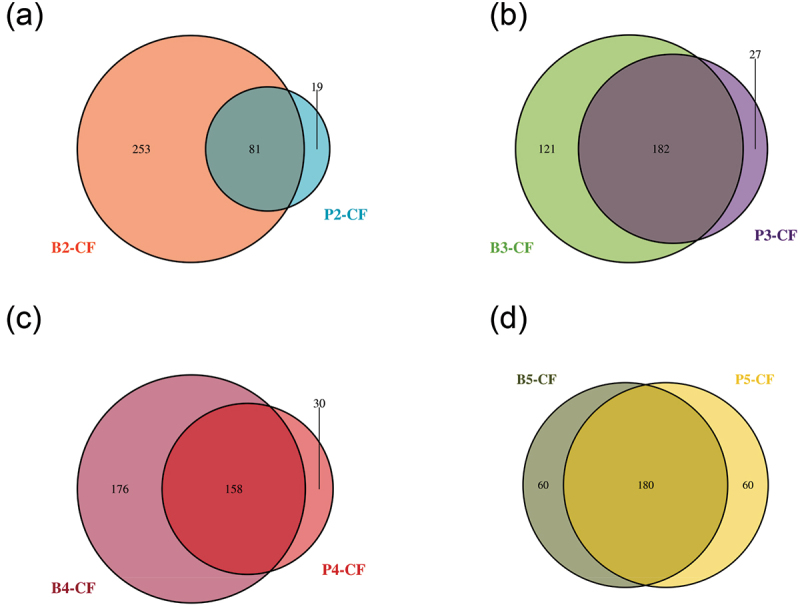
Venn diagrams depict the shared and unique OTUs within saliva and plaque samples at each state. (**a**) Venn diagrams at State 2, when only two lower primary incisors erupt. (**b**) Venn diagrams at State 3, when all eight primary incisors erupt. (**c**) Venn diagrams at State 4, when four primary first molars erupt. (**d**) Venn diagrams at State 5, when four primary second molars erupt. All OTUs are included.

**Figure A4.**
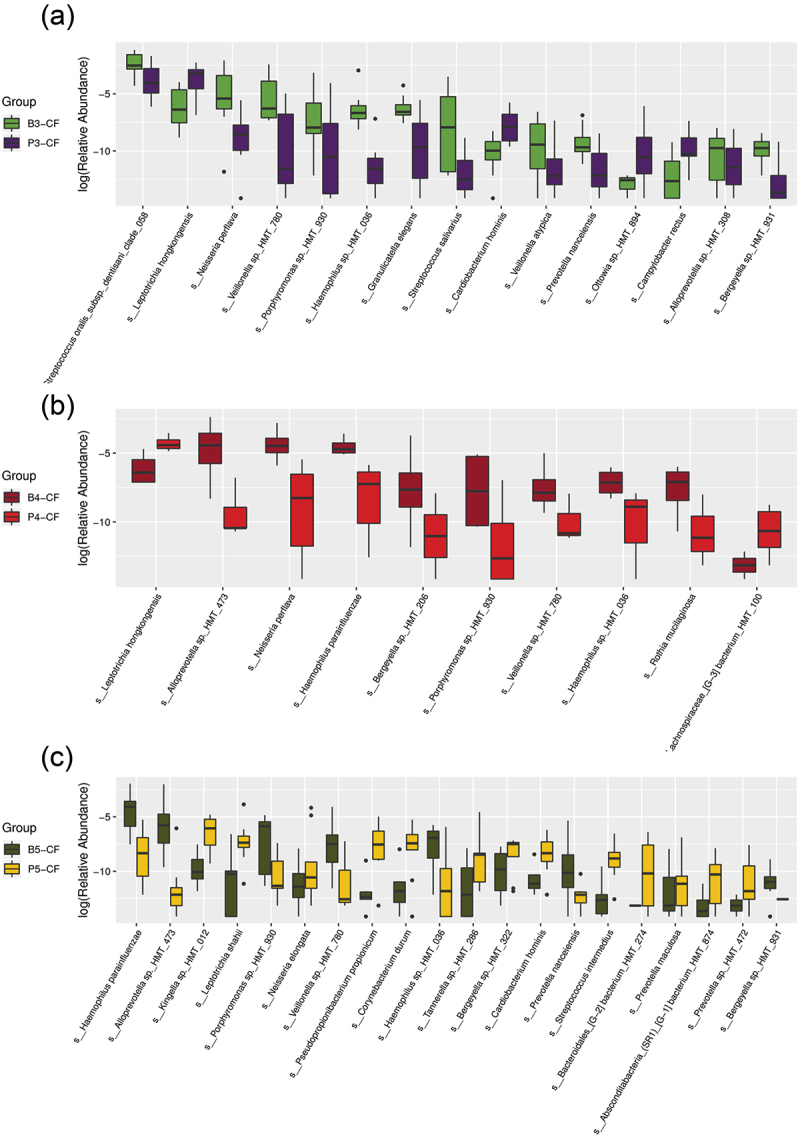
Comparison of OTU relative abundances at the species level between saliva and supragingival plaque. Differences in relative abundance are analyzed using the Wilcoxon rank-sum test, *P* < 0.05 is considered to reflect a significant difference. Not significant difference is not shown. (**a**) Comparison of relative abundances at State 3, when all eight primary incisors erupt. (**b**) Comparison of relative abundances at State 4, when four primary first molars erupt. (**c**) Comparison of relative abundances at State 5, when four primary second molars erupt.

## Appendix

**Table A1.** General information of participants and diversity estimation at 3% dissimilarity from the sequencing analysis

Sample	Gender	Age (month)	Clean reads		OTU^a^ num	
			Saliva	Plaque	Saliva	Plaque
CF1-S1	Female	4	62,802		139	
CF1-S2		8	58,662		153	
CF1-S3		16	60,221	64,125	144	124
CF1-S5		33	62,892	63,093	154	115
CF2-S3	Female	15	50,949	58,777	89	74
CF2-S4		22	57,710	48,022	199	98
CF2-S5		29	64,205		125	
CF3-S2	Male	7		32,442		85
CF3-S3		15	56,297		132	
CF3-S4		24	63,496		249	
CF3-S5		29	62,701	27,237	110	119
CF4-S2	Female	8	55,906		215	
CF4-S3		17	60,193	34,065	162	110
CF4-S5		34		24,756		120
CF5-S3	Male	16	57,965		92	
CF6-S4	Female	22		32,248		128
CF6-S5		31		57,405		140
CF7-S2	Female	8		32,191		46
CF7-S3		12	60,882	62,478	92	101
CF7-S4		19		60,500		111
CF7-S5		32		55,817		133
CF8-S3	Male	17		64,419		80
CF8-S4		25	40,404	22,033	94	91
CF8-S5		32	58,630	60,033	62	90
CF9-S3	Male	23		55,952		101
CF9-S5		34	57,304	59,251	120	95
CF10-S3	Male	13	56,209	55,233	85	59
CF10-S4		21		61,761		70
CF10-S5		37	64,344	40,078	92	102
CF12-S3	Male	16	56,017	61,005	111	94
CF12-S4		23	57,740	52,113	121	109
CF12-S5		36	64,393	52,421	125	113
CF13-S1	Female	7	57,844		266	
CF13-S2		9	58,174		183	
CF13-S3		14	49,080		132	
CF15-S1	Female	5	62,717		254	

^a^The operational taxonomic units (OTUs) are defined with 3% dissimilarity level.

**Table A2.** Relative abundance (%) of the OTUs at the phylum level per sample type and dentition state. Phyla ordered from the most to the least abundant in saliva at S1

Phylum	B-S1	B-S2	B-S3	B-S4	B-S5	P-S2	P-S3	P-S4	P-S5
*Firmicutes*	66.1	30.1	34.1	20.6	30.4	38.3	15.4	10.9	18.8
*Bacteroidetes*	7.7	12.1	14.9	18.7	15.7	7.3	13.6	13.8	8.0
*Actinobacteria*	6.9	5.4	5.0	2.1	4.8	27.3	7.9	7.5	7.7
*Proteobacteria*	4.0	12.1	21.7	29.3	26.5	16.8	24.4	26.8	21.1
*Saccharibacteria* (TM7)	0.2	1.1	1.1	0.4	1.0	0.4	1.0	0.2	2.5
*Fusobacteria*	0.1	4.9	6.3	4.1	4.5	1.5	19.8	17.0	19.4
*Absconditabacteria* (SR1)	0.0	0.0	0.0	0.1	0.0	0.0	0.0	0.3	0.1
*Gracilibacteria* (GN02)	0.0	0.0	0.2	0.1	0.0	0.0	0.0	0.2	0.1
*Spirochaetes*	0.0	0.0	0.0	0.0	0.0	0.0	0.0	0.0	0.2
*Synergistetes*	0.0	0.0	0.0	0.0	0.0	0.0	0.0	0.0	0.0

**Table A3.** Relative abundance (%) of 31 major genera at each dentition state in saliva and supragingival plaque

Genus	Relative abundance (%)								
	B-S1	B-S2	B-S3	B-S4	B-S5	P-S2	P-S3	P-S4	P-S5
*Streptococcus*	60.69	26.08	24.25	15.67	23.97	34.70	11.25	8.11	14.20
*Neisseria*	0.18	3.01	11.13	16.04	13.16	5.83	12.31	18.05	14.13
*Leptotrichia*	0.02	3.02	5.18	2.61	3.50	0.22	16.88	14.48	16.74
*Haemophilus*	2.55	4.95	6.07	6.61	11.50	10.58	1.87	0.94	1.03
*Granulicatella*	2.88	1.66	2.80	2.41	2.14	2.66	1.18	0.69	1.58
*Porphyromonas*	0.88	4.87	3.38	2.89	5.58	1.34	3.23	1.31	1.35
*Alloprevotella*	0.65	3.17	6.45	7.49	5.19	2.45	0.81	0.33	0.22
*Prevotella*	2.53	1.34	1.99	0.84	3.41	2.14	0.80	2.62	2.18
*Veillonella*	2.28	2.20	5.84	1.33	2.65	0.83	0.77	0.20	0.35
*Rothia*	5.47	2.22	2.76	0.80	2.68	27.17	0.40	0.07	0.34
*Capnocytophaga*	0.02	1.28	2.11	4.69	1.13	0.02	8.14	8.92	2.89
*Corynebacterium*	0.03	1.70	0.91	0.30	0.50	0.01	3.72	2.57	1.79
*Actinomyces*	0.14	1.32	1.19	0.34	1.06	0.00	3.49	1.38	4.12
*Fusobacterium*	0.04	1.89	1.09	1.47	1.02	1.29	2.95	2.55	2.70
*Bergeyella*	3.42	1.06	0.91	2.61	0.34	1.33	0.38	0.39	0.45
*Campylobacter*	0.39	0.36	0.53	0.17	0.55	0.31	1.42	0.42	0.62
*Saccharibacteria* (TM7) [G-6]	0.17	1.07	1.10	0.42	0.75	0.38	0.51	0.14	1.51
*Lautropia*	0.01	0.11	1.34	2.60	0.51	0.00	5.60	3.04	2.38
*Kingella*	0.01	0.24	0.92	2.65	0.58	0.00	2.15	3.18	2.15
*Cardiobacterium*	0.00	0.02	0.11	0.19	0.07	0.01	0.64	1.09	0.50
*Escherichia*	0.59	0.43	0.07	0.86	0.01	0.00	0.00	0.00	0.00
*Atopobium*	1.03	0.09	0.02	0.01	0.08	0.02	0.06	0.00	0.04
*Abiotrophia*	0.00	0.00	0.44	0.61	0.97	0.00	0.51	0.99	1.16
*Aggregatibacter*	0.00	0.01	0.13	0.02	0.12	0.00	0.11	0.01	0.28
*Brevibacterium*	0.13	0.06	0.02	0.09	0.01	0.04	0.01	0.01	0.00
*Gemella*	0.01	0.01	0.07	0.02	0.09	0.05	0.03	0.02	0.04
*Moraxella*	0.04	0.01	0.33	0.00	0.00	0.02	0.01	0.00	0.00
*Cutibacterium*	0.08	0.01	0.01	0.02	0.00	0.02	0.00	0.00	0.00
*Lactobacillus*	0.07	0.02	0.01	0.01	0.00	0.00	0.00	0.00	0.02
*Megasphaera*	0.05	0.01	0.01	0.02	0.01	0.00	0.03	0.00	0.01
*Catonella*	0.00	0.00	0.01	0.03	0.01	0.02	0.04	0.05	0.01

**Table A4.** Comparison of β-diversity of the saliva and supragingival plaque microbiome by the PERMANOVA test

	Saliva		Plaque	
Group	R^2^	*P* value	R^2^	*P* value
S1/S2	0.277	0.28		
S1/S3	0.222	0.007*		
S1/S4	0.471	0.015*		
S1/S5	0.249	0.015*		
S2/S3	0.077	0.692	0.223	0.023*
S2/S4	0.21	0.052	0.394	0.04*
S2/S5	0.116	0.376	0.219	0.018*
S3/S4	0.075	0.491	0.069	0.469
S3/S5	0.051	0.704	0.03	0.899
S4/S5	0.088	0.511	0.066	0.405

* *P* < 0.05
